# The Effect of Implanted Functional Electrical Stimulation on Gait Performance in Stroke Survivors: A Systematic Review

**DOI:** 10.3390/s21248323

**Published:** 2021-12-13

**Authors:** Gu Eon Kang, Rebecca Frederick, Brandon Nunley, Lawrence Lavery, Yasin Dhaher, Bijan Najafi, Stuart Cogan

**Affiliations:** 1 Department of Bioengineering, Erik Jonsson School of Engineering and Computer Science, The University of Texas at Dallas, Richardson, TX 75080, USA; rxf150330@utdallas.edu (R.F.); bsn200000@utdallas.edu (B.N.); sxc149830@utdallas.edu (S.C.); 2 Department of Plastic Surgery, The University of Texas Southwestern Medical Center, Dallas, TX 75390, USA; larry.lavery@utsouthwestern.edu; 3 Department of Physical Medicine and Rehabilitation, The University of Texas Southwestern Medical Center, Dallas, TX 75390, USA; yasin.dhaher@utsouthwestern.edu; 4 Interdisciplinary Consortium on Advanced Motion Performance (iCAMP), Michael E. DeBakey Department of Surgery, Baylor College of Medicine, Houston, TX 77030, USA; bijan.najafi@bcm.edu

**Keywords:** functional electrical stimulation, implant, gait, foot drop, stroke, stroke survivors, stroke rehabilitation

## Abstract

The emerging literature suggests that implantable functional electrical stimulation may improve gait performance in stroke survivors. However, there is no review providing the possible therapeutic effects of implanted functional electrical stimulation on gait performance in stroke survivors. We performed a web-based, systematic paper search using PubMed, the Cochrane Library, and EMBASE. We limited the search results to human subjects and papers published in peer-reviewed journals in English. We did not restrict demographic or clinical characteristics. We included 10 papers in the current systematic review. Across all included studies, we found preliminary evidence of the potential therapeutic effects of functional electrical stimulation on walking endurance, walking speed, ankle mobility, and push-off force in stroke survivors. However, due to the heterogeneity between the included studies, small sample size, and lack of randomized controlled trials, more studies are critically needed to confirm whether implanted functional electrical stimulation can improve gait performance in stroke survivors.

## 1. Introduction

In the United States (US), nearly 800,000 people experience a stroke each year [1], and approximately 4% of the US population will have experienced a stroke by 2030 [2]. Globally, stroke is ranked as the second leading cause of mortality, with more than 5 million deaths attributed to stroke in 2016 [3]. Stroke is also ranked as the second most common cause of disability worldwide, accounting for more than 116 million disability-adjusted life-years in 2016 [3].

Approximately 80% of strokes are ischemic, caused by blood clots blocking blood flow to the brain, and 20% are hemorrhagic, caused when a blood vessel leaks or ruptures [4]. The treatment of ischemic stroke is time-sensitive and requires thrombolytic therapy (delivered intravenously). However, for hemorrhagic stroke, thrombolytic therapy is contraindicated. Patients with hemorrhagic stroke commonly have inferior neurological outcomes and a lower survival rate within the first 30 days post-stroke than those with ischemic stroke [5]. Treatment of either type of stroke is time-sensitive, and if recognized early, is treatable.

It has been estimated that more than 70% of people who survive a stroke sustain some feature of gait impairment post-stroke [6]. This gait impairment results in increased risk of falls in stroke survivors [7]. Indeed, studies have reported that almost 50% of stroke patients fall during inpatient rehabilitation [8], and that up to 73% of stroke survivors fall during the first six months after rehabilitation [9]. The high risk of falls in stroke survivors, in turn, leads to serious problems such as fracture injuries [10], an onset of a fear of falling [11], and further restrictions on activity and mobility [12,13].

One expression of gait impairment in stroke is the significant reduction in distal lower limb strength [14]. While walking, this reduction results in the inability to properly dorsiflex the ankle joint immediately after the push-off and safely clear the ground, traditionally known as ”foot drop” (i.e., difficulty in lifting up the front part of the foot) [15].

Functional electrical stimulation using surface electrodes is often used as a rehabilitation technique to help manage foot drop and improve gait performance. Functional electrical stimulation provides electrical currents through transcutaneous electrodes to stimulate peripheral nerves that activate the ankle dorsiflexor muscles [16]. A tilt sensor or in-shoe sensor is commonly used to wirelessly control the stimulation. After the initial introduction of this approach [17], functional electrical stimulation has been continuously studied, developed, and utilized to restore lower limb functions in stroke survivors [18]. However, recent human-centric examinations reported some limitations related to the surface-based functional electrical stimulation such as changes in skin resistance, skin irritation, and a physical and cosmetic discomfort regarding the external device [19,20,21].

In an attempt to address the aforementioned limitations, implanted functional electrical approaches were developed. Since the official approval in Germany in 2007 for stroke survivors [22], several trials investigated the effect of implanted functional electrical stimulation on treating gait impairment in stroke survivors. However, to date, there is no published review of the therapeutic effect of the implementable approach on gait impairment. Accordingly, we aimed to systematically review the effect of implanted functional electrical stimulation on gait performance in stroke survivors. The research question posed in this review was: Does implanted functional electrical stimulation improve gait performance in stroke survivors?

## 2. Materials and Methods

### 2.1. Search Strategy

In this systematic review, we followed the Preferred Reporting Items for Systematic Reviews and Meta-Analyses (PRISMA) guidelines [23]. We performed a web-based, systematic paper search using PubMed, the Cochrane Library, and EMBASE for papers published before 5 October 2021. The keywords that were used for the systematic paper search are as follows: “electrical stimulation”, “foot drop stimulator”, “gait”, “gait parameters”, “gait speed”, “gait stability”, “gait initiation”, “gait kinematics”, “gait kinetics”, “stroke survivors”, and “stroke rehabilitation” (see Table 1 for the full search strategy). We limited search results to peer-reviewed journal papers published in English, and studies with human subjects.

### 2.2. Study Selection

We included papers that reported the therapeutic efficacy of implanted functional electrical stimulation on gait performance in stroke survivors with foot drop as randomized controlled trials or observational studies. Outcomes of this systematic review were quantitative measures of gait including spatiotemporal parameters (e.g., gait speed), gait stability (e.g., stride-to-stride asymmetry, movement smoothness, obstacle negotiation), and gait kinetics (e.g., plantar load). We excluded papers that provided a qualitative description of gait performance using subjective methods. Additionally, we excluded conference abstracts, editorial papers, case studies, and review papers from the final study selection.

Two independent reviewers (Rebecca Frederick and Brandon Nunley) conducted an initial screening of the searched papers based on title and abstract. Another reviewer (Gu Eon Kang) independently provided the deciding vote on disagreements (*n* = 43).

## 3. Results

### 3.1. Search Results

**Figure 1:**
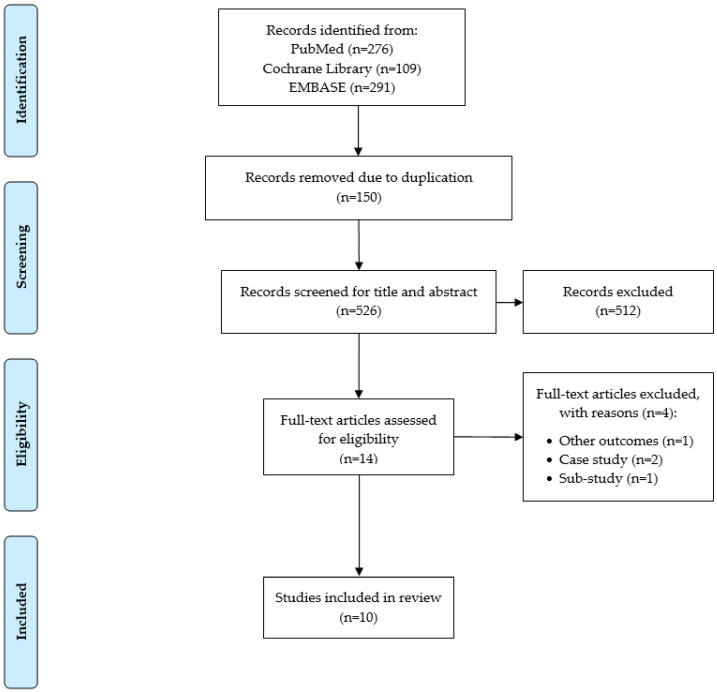
PRISMA consort diagram for paper selection.

The flow diagram for study selection is shown in Figure 1. We identified 676 papers through PubMed, the Cochrane Library, and EMBASE. After obtaining the initial record of papers, we excluded 150 papers due to duplication using a reference management software (EndNote, Philadelphia, PA, USA). We screened the title and abstract for the remaining 526 papers and excluded 512 papers that did not report the efficacy of implantable functional electrical stimulation on gait performance in stroke survivors. We excluded another 4 papers after assessing eligibility criteria based on full-text review. Consequently, we included 10 papers in the current systematic review [24,25,26,27,28,29,30,31,32,33].

### 3.2. Study Design and Countries

Our findings are summarized in Table 2. All included studies were published in the last 15 years and were conducted in European countries (*n* = 5 in Germany; *n* = 4 in the Netherlands; *n* = 1 in Denmark). Two studies (20.0%) were randomized controlled trials and eight studies (80.0%) were single arm trials. The vast majority of the included studies (90.0%) followed the study participants longitudinally: Follow-up periods ranged from 1–12 months. One study reported an immediate effect of the implanted functional electrical stimulation (i.e., ON vs. OFF), and did not follow their study participants over the longitudinal course.

### 3.3. Characteristics of Stroke Survivors

The total number of recruited participants across studies was 161 at the baseline. Dropout rates at the conclusion of these studies ranged between 0% and 26.7%. The mean age of the recruited stroke survivors was early to mid-50s for the vast majority of the studies (80.0%) and late 40s for the remaining studies (20.0%). Across all studies, slightly more than half of the recruited participants (*n* = 81; 50.3%) had right hemiplegia. Types of strokes included in the studies were ischemic (*n* = 85), hemorrhage (*n* = 50), and unknown (*n* = 1). One participant in Bucklitsch et al. (2019) and four participants in Buentjen et al. (2019) had foot drop due to multiple sclerosis. Kottink et al. (2007 and 2012) and Ernst et al. (2013) did not report types of strokes. The time since the onset of stroke ranged, approximately, between 5 and 15 years.

### 3.4. Intervention

Eight studies (80.0%) utilized the ActiGait foot drop stimulator (Otto Bock, Duderstadt, Germany), which consists of an implantable 4-channel peroneal nerve stimulator, 12-contact electrode cuff, an external control unit, and a heel switch to activate the stimulator (see Burridge et al. (2007) for more details). For the ActiGait foot drop stimulator, the location of the implant was proximal to the common peroneal nerve’s bifurcation into the deep and superficial branches of the nerve that innervate the ankle dorsiflexors.

Two studies (20.0%; Kottink et al. (2007 and 2012)) utilized a foot drop stimulator (it was not reported whether the stimulator was commercially available or was developed by the authors), which consisted of an implantable 2-channel peroneal nerve stimulator, bipolar intraneural electrodes, an external transmitter, a heel switch to activate the transmitter (see Kottink et al. (2007) for more details). The location of the implant was within the epineurium of the superficial peroneal nerve.

For most of studies (*n* = 7), surgical procedures for implantation were performed under general anesthesia. In four studies (40.0%), adverse events including minor wound infection, delayed wound healing, hematoma, bleeding, and neurodermatitis were reported. One study reported that a participant died but it was not related to the implant. Six studies (60.0%) did not report adverse events.

### 3.5. Gait Outcomes

Four studies (40.0%) reported walking endurance based on the 6-min walk test (with the exception of one study employing the 4-min walk test). Eight studies (80.0%) reported walking speed at a comfortable pace and/or fast pace. Among the eight studies that reported walking speed, two studies reported other spatiotemporal gait parameters such as stride length, cadence, double support phase, and single support phase. Two studies (20.0%) reported outcomes related to gait stability: step width, step length asymmetry, and effective foot length. Four studies (40.0%) reported joint kinematics, such as range of motion, in the hip, knee, or ankle during walking. Four studies (40.0%) reported kinetic outcomes during gait: plantar pressure, ground reaction forces, and ankle power. Two studies reported other outcomes including objective measures of physical activity level and the duration of timed up and go.

### 3.6. Summary of Key Results

Across the four studies that reported endurance measures, 4-min or 6-min walk distances were improved at follow-up. Significant improvement was reported by Martin et al. (2016) at 6-weeks, and by Burridge et al. (2007) at 15-months. Some studies did not report *p*-values for their walking endurance data.

In terms of walking speed and other spatiotemporal gait parameters, overall, most studies reported improvements (either significant or non-significant) in these outcomes. Significant improvements were reported by Burridge et al. (2007) at 90-day and 15-month assessments, by Martin et al. (2016) after 6-weeks, and by Buenijen et al. (2019) at 12-months. Gait stability was also improved with implanted functional electrical stimulation, but the improvements were non-significant.

As for joint kinematics, Daniilidis et al. (2017) and Berenpas et al. (2018) reported significant improvements in joint kinematics such as sagittal knee and ankle angles. Daniilidis et al. (2017) also reported significant improvements in gait kinetics (peak vertical ground reaction force). 

## 4. Discussion

In this paper, we aimed to provide a systematic review of the effect of implanted functional electrical stimulation on gait performance in stroke survivors. We found a total of 10 papers that were within inclusion and exclusion criteria. Preliminary findings from this examination suggest stroke survivors may express some level of improvement in walking endurance, walking speed, ankle mobility, and push-off after receiving functional electrical stimulation from an implanted stimulator.

In the context of assessing the efficacy of implanted functional electrical stimulation for ankle control, no randomized controlled trials were identified. Most of the included studies used a single arm trial design. Since the primary purpose of using implanted functional electrical stimulation would be an alternative to ankle–foot orthoses and surface-based functional electrical stimulation, there is a critical need to conduct randomized controlled trials comparing the therapeutic effects of implanted functional electrical stimulation and conventional first-line treatments.

Our review also highlights a few issues regarding study participants: (1) Small sample size, (2) varied demographic (e.g., age) and clinical characteristics (time since stroke), and (3) varied follow-up time points. Furthermore, across all studies, we found a lack of measuring possible common covariates that could mitigate the effect of the experimental intervention such as frailty, fear of falling, and cognitive status [34,35].

Another important limitation of the included papers is the heterogeneity in the type of stroke. Several previous papers reported mixed results about the effect of other rehabilitation techniques on functional outcomes between ischemic and hemorrhagic patients [36,37]. However, based on our systematic review, we found that the reported effects of implanted functional electrical stimulation may have been confounded by different types of strokes included in the patient population studied. It will be important to investigate the influence of the type and severity of stroke on functional outcomes after being treated with implanted functional electrical stimulation.

We also found another important limitation regarding the stimulation specifications (amplitude, frequency, duration), which was found only in half of the included studies. As these specifications may have affected the results of gait performance tested in the included studies, it will be critical to clearly report in future studies.

We also found issues regarding gait outcome measures. In terms of walking endurance tests (i.e., either 4-min walk distance vs. 6-min walk distance), although it may be too early to determine based on the limited number of included studies, given the validity and popularity [38], the 6-min walk test may better reflect walking endurance in stroke survivors. Furthermore, gait speed during level short-distance walking that was reported in 80% of the included studies may not provide a comprehensive view of gait performance in stroke survivors because this walking condition may not best represent gait performance in natural circumstances. To address this issue, we recommend investigating other gait outcomes such as gait stability and gait initiation under various walking conditions (e.g., dual-task walking, changing directions during walking).

Additionally, although a few studies reported ankle mobility during walking, we noticed heterogeneity in the reported outcome variables for ankle mobility. A direct quantitative measure of foot drop, namely, changes in sagittal ankle angle around push-off and the associated compensatory movements like hip hiking is lacking. Further, no studies addressed other types of gait dysfunction besides foot drop, i.e., dysfunction in muscle groups other than the dorsiflexors.

## 5. Conclusions

Based on our systematic review, we found preliminary evidence of the therapeutic effects of implanted functional electrical stimulation on gait performance in stroke survivors. However, it seems premature to positively assert the therapeutic benefit of implantable functional electrical stimulation due to the limited number of examinations and the corresponding design limitations that were identified in this literature. Additional studies are needed to further investigate the therapeutic effects of implanted functional stimulation in stroke survivors. Furthermore, future work will need to evaluate the effects of implanted functional electrical stimulation not only for correcting foot drop (evoking the contraction of dorsiflexor muscles), but also for addressing dysfunction in other muscles or muscle groups in the lower limb that can contribute to overall gait dysfunction following stroke.

## Figures and Tables

**Table 1:** Full search strategy.

PubMed Search	
	“Electric Stimulation” [MeSH Terms] OR “Electric Stimulation Therapy” [MeSH Terms] OR “Electrical Stimulation” [Text Word] OR “Foot Drop Stimulator” [Text Word] OR “Peroneal Nerve Stimulator” [Text Word]
AND	“Walking” [MeSH Terms] OR “Gait” [MeSH Terms] OR “Locomotion” [MeSH Terms] OR “Gait Parameters” [Text Word] OR “Gait Speed” [Text Word] OR “Gait Stability” [Text Word] OR “Gait Initiation” [Text Word] OR “Gait Kinematics” [Text Word] OR “Gait Kinetics” [Text Word] OR “Ankle Power” [Text Word] OR “Mobility” [Text Word]
AND	“Stroke” [MeSH Terms] OR “Stroke Rehabilitation” [MeSH Terms] OR “Stroke Rehabilitation” [Text Word] OR “Stroke Survivors” [Text Word] OR “Post Stroke” [Text Word] OR “Poststroke” [Text Word] OR “Post-stroke” [Text Word]
**Cochrane Library Search**	
	MeSH descriptor: [Electric Stimulation] explode all trees OR MeSH descriptor: [Electric Stimulation Therapy] explode all trees OR (Foot Drop Stimulator):ti,ab,kw OR (Peroneal Nerve Stimulator):ti,ab,kw
AND	MeSH descriptor: [Gait] explode all trees OR MeSH descriptor: [Gait] explode all trees OR MeSH descriptor: [Locomotion] explode all trees OR (Gait Parameters):ti,ab,kw OR (Gait Speed):ti,ab,kw OR (Gait Stability):ti,ab,kw OR (Gait Initiation):ti,ab,kw OR (Gait Kinematics):ti,ab,kw OR (Ankle Power):ti,ab,kw OR (Gait Kinetics):ti,ab,kw OR (Mobility):ti,ab,kw OR
AND	MeSH descriptor: [Stroke] explode all trees OR MeSH descriptor: [Stroke Rehabilitation] explode all trees OR (Stroke Survivor):ti,ab,kw OR (Post Stroke):ti,ab,kw OR (Post-stroke):ti,ab,kw OR (Poststroke):ti,ab,kw
**EMBASE Search**	
	‘Electrostimulation’/exp OR Electrostimulation
AND	‘Gait’/exp OR Gait OR ‘Walking’/exp OR Walking OR ‘Locomotion’/exp OR Locomotion)
AND	‘Stroke’/exp OR Stroke

**Table 2:** Characteristics of studies included for full review.

Study Country Study Design	Participant Characteristics Follow-Up	Electrical Stimulator Stimulation Specification (Voltage, Current, Phase Duration, Frequency)	Surgical Procedure Surgery-Related Adverse Events	Gait Outcomes	Key Results
Burridge et al. 2007 Denmark Single arm trial	Baseline *n* = 15 (4 men; 11 women) Mean age (years) = 56.8 ± 7.6 Side of hemiplegia (7 right; 8 left) Type of stroke (8 ischemic; 5 hemorrhage; 1 unknown) Time since stroke (years) = 4.9 ± 1.9 90-day (*n* = 13) 15-month (*n* = 13)	ActiGait (4-channel peroneal nerve stimulator; 12-contact electrode cuff; an external control unit; a heel switch) Stimulation specification not reported	Spinal anesthesia (*n* = 8) General anesthesia (*n* = 7) Implant location (the electrode cuff): Just proximal to the common peroneal nerve’s bifurcation into the deep and superficial branches to the tibialis anterior and the peronei muscles Stimulator location: 1 or 2 sutures to the lateral femoral fascia A longitudinal incision along the tendon of the biceps femoris Second incision: The lateral side of the femur, posterior to the location for the simulator body Minor wound infections (*n* = 2; treated with antibiotics) Delayed wound healing (*n* = 1)	4-min walk distance Walking speed (20-m)	Compared to baseline: At 90-day 4-min walk distance ↑ (*p* ≥ 0.05) Walking speed ↑ (*p* < 0.05) At 15-month 4-min walk distance ↑ (*p* < 0.05) Walking speed ↑ (*p* < 0.05)
Kottink et al. 2007 The Netherlands Randomized controlled trial	Intervention group (IG) Baseline *n* = 14 (10 men; 4 women) Mean age (years) = 55.2 ± 11.4 Side of hemiplegia (7 right; 7 left) Type of stroke (not reported) Time since stroke (years) = 9.1 ± 9.3 Control group (CG) Baseline *n* = 15 (10 men; 5 women) Mean age (years) = 52.9 ± 9.9 Side of hemiplegia (9 right; 6 left) Type of stroke (not reported) Time since stroke (years) = 9.1 ± 9.3 4-week (*n* = 14 in IG; *n* = 12 in CG) 8-week (*n* = 14 in IG; *n* = 12 in CG) 12-week (*n* = 14 in IG; *n* = 11 in CG) 26-week (*n* = 14 in IG; *n* = 11 in CG)	IG 2-channel peroneal nerve stimulator; an external transmitter; a foot switch Stimulation pulse: Asymmetric biphasic charge-balanced-waveform (30 Hz); No other information reported CG Ankle–foot orthosis, orthopedic shoes, or no device	Spinal or general anesthesia (number of participants not reported) Implant location: One electrode was placed under the epineurium of the superficial peroneal nerve The other electrode was placed under the epineurium of the deep peroneal nerve The receiver body was placed ina subcutaneous pocket Incision: Approximately 50 mm along the common peroneal nerve Adverse events not reported	6-min walk distance Walking speed (10-m) Other outcomes: 5-day activity level	Compared to baseline (IG): At 4-week No 6-min walk distance results Walking speed ↓ (*p*-value not reported) No activity level results At 8-week No 6-min walk distance results Walking speed ↑ (*p*-value not reported) No activity level results At 12-week 6-min walk distance ↑ (*p*-value not reported) Walking speed ↑ (*p*-value not reported) No activity level results At 26-week 6-min walk distance ↑ (*p*-value not reported) Walking speed ↑ (*p* < 0.05) % Duration of walking ↓ (*p* < 0.05) % Duration of standing ↓ (*p* ≥ 0.05) % Duration of sitting/lying ↑ (*p* < 0.05) Note: At every follow-up, IG had greater 6-min walk distance and walking speed than CG.
Kottink et al. 2012 The Netherlands Randomized controlled trial	IG Baseline *n* = 10 (7 men; 3 women) Mean age (years) = 55.6 ± 13.2 Side of hemiplegia (4 right; 6 left) Type of stroke (not reported) Time since stroke (years) = 9.0 ± 10.0 CG Baseline *n* = 13 (8 men; 5 women) Mean age (years) = 53.3 ± 10.6 Side of hemiplegia (5 right; 8 left) Type of stroke (not reported) Time since stroke (years) = 6.2 ± 4.8 26-week (*n* = 9 in IG; *n* = 12 in CG) Note: Participants are a subset of Kottink et al. (2007).	Same as Kottink et al. (2007)	Surgical procedure is same as Kottink et al. (2007)	Walking speed (10-m) Stride time Stride width Step length (paretic side and non-paretic side) Stance phase (paretic side and non-paretic side) Double support phase (paretic side and non-paretic side) Single support phase (paretic side and non-paretic side) Sagittal hip range of motion Sagittal knee range of motion Sagittal ankle range of motion	Compared to baseline (IG): At 26-week Walking speed ↑ (*p* ≥ 0.05) Stride time ↓ (*p* < 0.05) Stride width no change Step length (paretic side) ↓ (*p* ≥ 0.05) Step length (non-paretic side) ↑ (*p* ≥ 0.05) Stance phase (paretic side) ↓ (*p* ≥ 0.05) Stance phase (non-paretic side) ↓ (*p* ≥ 0.05) Double support phase (paretic side) ↓ (*p* ≥ 0.05) Double support phase (non-paretic side) ↓ (*p* ≥ 0.05) Single support phase (paretic side) ↑ (*p* ≥ 0.05) Single support phase (non-paretic side) ↑ (*p* ≥ 0.05) Sagittal hip range of motion ↑ (*p* ≥ 0.05) Sagittal knee range of motion ↓ (*p* ≥ 0.05) Sagittal ankle range of motion ↓ (*p* ≥ 0.05)
Ernst et al. 2013 Germany Single arm trial	Baseline *n* = 5 (3 men; 2 women) Mean age (years) = 47.2 (standard deviation not reported) Side of hemiplegia (3 right; 2 left) Type of stroke (not reported) Time since stroke (years) = 5.6 (standard deviation not reported) 6-week (*n* = 5) 12-week (*n* = 5)	ActiGait (4-channel peroneal nerve stimulator; 12-contact electrode cuff; an external control unit; a heel switch) Stimulation specification not reported	General anesthesia (*n* = 5) Surgical procedure is same as Burridge et al. (2007) Serious adverse event (*n* = 1; a hematoma around the distal incision and bleeding after removing stitches) Post-surgical lymphoedema around the proximal incision (*n* = 1); Both participants were included in the study	6-min walk distance Walking speed (10-m) Sagittal ankle angle	Compared to baseline: At 6-week 6-min walk distance ↑ (*p*-value not reported) Walking speed ↑ (*p*-value not reported) Sagittal ankle angle not assessed At 12-week 6-min walk distance ↑ (*p*-value not reported) Walking speed ↑ (*p*-value not reported) Sagittal ankle angle at heel strike and during loading-phase ↓ (*p* < 0.05) Sagittal ankle angle at mid-stance and during pre-swing ↓ (*p* ≥ 0.05)
Schiemanck et al. 2015 The Netherlands Single arm trial	Baseline *n* = 10 (5 men; 5 women) Mean age (years) = 47.4 ± 14.5 Side of hemiplegia (4 right; 6 left) Type of stroke (8 ischemic; 2 hemorrhage) Time since stroke (years) = 5.6 ± 2.4 2-week (*n* = 8) 8-week (*n* = 8) 26-week (*n* = 8)	ActiGait (4-channel peroneal nerve stimulator; 12-contact electrode cuff; an external control unit; a heel switch) Stimulation specification not reported Note: Participants used ankle–foot orthosis along with the electrical stimulation.	Surgical procedure not reported Adverse events not reported	Ankle plantarflexion power (15-m)	Compared to baseline: Ankle plantarflexion power ↑ at all follow-ups (*p*-value not reported)
Martin et al. 2016 Germany Single arm trial	Baseline *n* = 27 (14 men; 13 women) Mean age (years) = 51.0 ± 11.6 Side of hemiplegia (15 right; 12 left) Type of stroke (21 ischemic; 6 hemorrhage) Time since foot drop (years) = 5.2 ± 4.8 6-week (*n* = 27)	ActiGait (4-channel peroneal nerve stimulator; 12-contact electrode cuff; an external control unit; a heel switch) Stimulation pulse: 1 mA, 20–35 Hz, 45–330 µs pulse width Ankle dorsiflexion tested with maximum 6 volts, 10 mA, 2.5 Hz for electrode placement	General anesthesia (*n* = 27) Other surgical procedure is same as Burridge et al. (2007) Nerve injury (*n* = 2; reoperation was performed) Wound healing disorder (*n* = 8) Neurodermatitis and infection (*n* = 1)	6-min walk distance Walking speed (20-m) Other outcomes: Duration of timed up and go	Compared to baseline: At 6-week 6-min walk distance ↑ (*p* < 0.05) Walking speed ↑ (*p* < 0.05) Duration of timed up and go ↓ (*p* < 0.05)
Daniilidis et al. 2017 Germany Single arm trial	Baseline *n* = 18 (12 men; 6 women) Mean age (years) = 51.3 ± 8.4 Side of hemiplegia (9 right; 9 left) Type of stroke (13 ischemic; 5 hemorrhage) Time since stroke (years) = 7.2 ± 5.2 12-month (*n* = 18)	ActiGait (4-channel peroneal nerve stimulator; 12-contact electrode cuff; an external control unit; a heel switch) Stimulation pulse: 1.1 mA, 20–30 Hz, 70 μs pulse width (initially; pusle width and timing were adjusted for each patient throughout the study) Ankle dorsiflexion tested with maximum 6 volts, 10 mA, 2.5 Hz for electrode placement	General anesthesia (*n* = 18) Other surgical procedure is same as Burridge et al. (2007) Adverse events not reported	Walking speed (walking distance not reported) Stride length Cadence Double support phase Ankle dorsiflexion angle Vertical ground reaction force Anterior-posterior ground reaction force	Compared to baseline: At 12-month Walking speed ↑ (*p* ≥ 0.05) Stride length ↑ (*p* ≥ 0.05) Cadence ↑ (*p* ≥ 0.05) Double support phase ↑ (*p* ≥ 0.05) Ankle dorsiflexion angle ↑ (*p* < 0.05) Peak vertical ground reaction force ↑ (*p* < 0.05) Peak anterior-posterior ground reaction force ↑ (*p* ≥ 0.05)
Berenpas et al. 2018 The Netherlands Single arm trial	Baseline *n* = 19 (14 men; 5 women) Mean age (years) = 54.4 ± 12.3 Side of hemiplegia (8 right; 11 left) Type of stroke (14 ischemic; 5 hemorrhage) Time since stroke (years) = 5.0 ± 3.7 2-week (*n* = 19) 8-week (*n* = 19) 26-week (*n* = 19)	ActiGait (4-channel peroneal nerve stimulator; 12-contact electrode cuff; an external control unit; a heel switch) Stimulation specification not reported Note: Participants use ankle–foot orthosis along with the electrical stimulation.	Surgical procedure is same as Burridge et al. (2007) Anesthesia information not reported Peroneal nerve damage (*n* = 1) Death (*n* = 1; Not related to surgery) Severe calf muscle clonus in reaction to electrical stimulation (*n* = 1)	Walking speed (10-m) Step length asymmetry Maximum hip flexion angle and velocity Maximum knee flexion angle and velocity Maximum knee extension velocity Maximum ankle plantarflexion angle, velocity, and power	Compared to 2-week (no baseline data were provided except walking speed): At 8-week Walking speed ↑ (*p* ≥ 0.05) Step length asymmetry ↓ (*p* ≥ 0.05) Maximum hip flexion angle ↑ (*p* < 0.05) Maximum hip flexion velocity ↓ (*p* ≥ 0.05) Maximum knee flexion angle ↑ (*p* < 0.05) Maximum knee flexion velocity ↑ (*p* ≥ 0.05) Maximum knee extension velocity ↑ (*p* < 0.05) Maximum ankle plantarflexion angle ↑ (*p* ≥ 0.05) Maximum ankle plantarflexion velocity ↑ (*p* ≥ 0.05) Maximum ankle plantarflexion power ↑ (*p* ≥ 0.05) At 26-week Walking speed ↑ (*p* ≥ 0.05) Step length asymmetry ↑ (*p* ≥ 0.05) Maximum hip flexion angle ↑ (*p* < 0.05) Maximum hip flexion velocity ↑ (*p* ≥ 0.05) Maximum knee flexion angle ↑ (*p* < 0.05) Maximum knee flexion velocity ↑ (*p* ≥ 0.05) Maximum knee extension velocity ↑ (*p* < 0.05) Maximum ankle plantarflexion angle ↓ (*p* ≥ 0.05) Maximum ankle plantarflexion velocity ↑ (*p* ≥ 0.05) Maximum ankle plantarflexion power ↑ (*p* ≥ 0.05)
Bucklitsch et al. 2019 Germany Single arm trial	Baseline *n* = 8 (2 men; 6 women) Mean age (years) = 58.1 ± 6.3 Side of hemiplegia (2 right; 6 left) Type of stroke (6 ischemic; 1 hemorrhage) Time since stroke (years) = 15.3 ± 10.6 Immediate effect (*n* = 8) Note: This study included multiple sclerosis (*n* = 1)	ActiGait (4-channel peroneal nerve stimulator; 12-contact electrode cuff; an external control unit; a heel switch) Stimulation specification not reported	General anesthesia (*n* = 8) Implant location (the electrode cuff): Around the peroneal nerve. Implant location (the stimulator): Lateral femoral fascia 4- to 5-cm incision above the knee Curved incision at the upper leg Adverse events not reported	Plantar pressure Step width Effective foot length Double support phase	Compared to baseline When the stimulation was on Plantar pressure ↓ (*p* ≥ 0.05) Step width ↓ (*p* ≥ 0.05) Effective foot length ↑ (*p* < 0.05) Double support phase ↓ (*p* ≥ 0.05)
Buentjen et al. 2019 Germany Single arm trial	Baseline *n* = 45 (24 men; 21 women) Mean age (years) = 52.0 ± 12.0 Side of hemiplegia (26 right; 19 left) Type of stroke (15 ischemic; 26 hemorrhage) Time since stroke (years) = 5.9 ± 6.1 1-day (*n* = 33) 3-month (*n* = 33) 12-month (*n* = 33) Note: This study included multiple sclerosis (*n* = 4)	ActiGait (4-channel peroneal nerve stimulator; 12-contact electrode cuff; an external control unit; a heel switch) Simulation pulse: No voltage information reported, 30–250 µs, 15–45 Hz	General anesthesia (*n* = 45) Surgical procedure is same as Burridge et al. (2007) Adverse events not reported	Maximum and comfortable gait speed (10-m walkway)	Compared to 1-day (no baseline data were provided except walking speed): At 3-month Maximum gait speed ↑ (*p* < 0.05) Comfortable gait speed ↑ (*p* ≥ 0.05) At 12-month Maximum gait speed ↑ (*p* < 0.05) Comfortable gait speed ↑ (*p* ≥ 0.05)

## Data Availability

Not applicable.
